# AMST: Alignment to Median Smoothed Template for Focused Ion Beam Scanning Electron Microscopy Image Stacks

**DOI:** 10.1038/s41598-020-58736-7

**Published:** 2020-02-06

**Authors:** Julian Hennies, José Miguel Serra Lleti, Nicole L. Schieber, Rachel M. Templin, Anna M. Steyer, Yannick Schwab

**Affiliations:** 1 European Molecular Biology Laboratory (EMBL), Cell Biology and Biophysics Unit, Heidelberg, 69117 Germany; 2 Collaboration for joint PhD degree between EMBL and Heidelberg University, Faculty of Biosciences, Heidelberg, 69117 Germany; 3 Max Planck Institute of Experimental Medicine, Electron Microscopy Core Unit, Department of Neurogenetics, 37075 Göttingen, Germany

**Keywords:** Data processing, Image processing, Machine learning, Software

## Abstract

Alignment of stacks of serial images generated by Focused Ion Beam Scanning Electron Microscopy (FIB-SEM) is generally performed using translations only, either through slice-by-slice alignments with SIFT or alignment by template matching. However, limitations of these methods are two-fold: the introduction of a bias along the dataset in the **z**-direction which seriously alters the morphology of observed organelles and a missing compensation for pixel size variations inherent to the image acquisition itself. These pixel size variations result in local misalignments and jumps of a few nanometers in the image data that can compromise downstream image analysis. We introduce a novel approach which enables affine transformations to overcome local misalignments while avoiding the danger of introducing a scaling, rotation or shearing trend along the dataset. Our method first computes a template dataset with an alignment method restricted to translations only. This pre-aligned dataset is then smoothed selectively along the **z**-axis with a median filter, creating a template to which the raw data is aligned using affine transformations. Our method was applied to FIB-SEM datasets and showed clear improvement of the alignment along the **z**-axis resulting in a significantly more accurate automatic boundary segmentation using a convolutional neural network.

## Introduction

**Figure 1:**
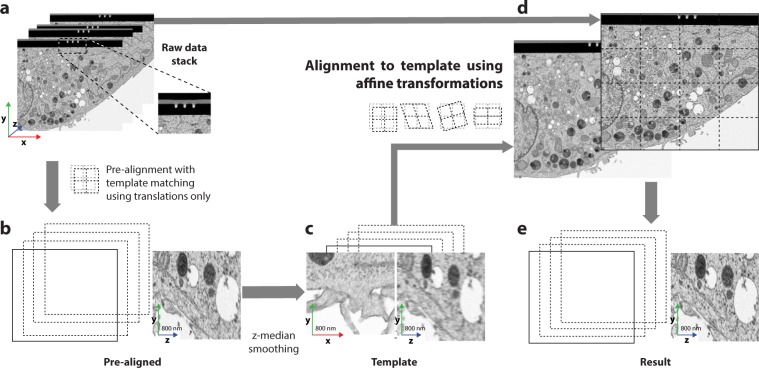
Workflow of Alignment to Median-Smoothed Template (AMST). The raw data (**a**) is used to generate a pre-alignment (**b**). The pre-aligned data is smoothed along the **z**-axis by a median filter which yields the template dataset (**c**) to which the raw data (**a**) is aligned using affine transformations, i.e. translation, shearing, rotation, and scaling (**d**), thus generating the final result (**e**). The inset in (**a**) shows markings in the platinum coating used for auto-focus and auto-stigmation operations during image acquisition. These can be used for pre-alignment by TM. All images are from the HeLa dataset (EMPIAR-10311).

**Figure 2:**
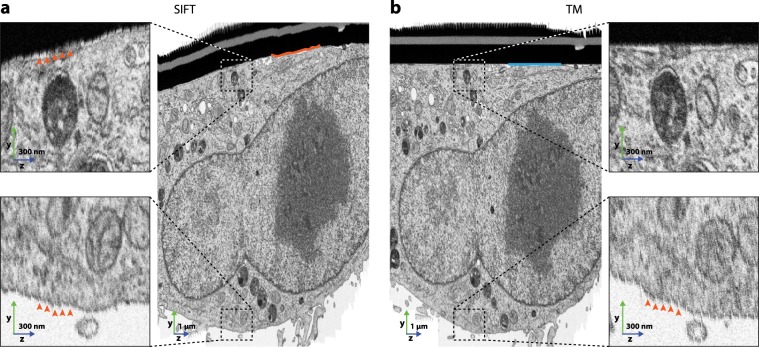
Qualitative comparison of (**a**) alignment by SIFT and (**b**) alignment by template matching (TM) using the HeLa dataset (EMPIAR-10311). The alignment of the platinum layer (appearing as black bar above the cell) is a good indicator for the preservation of large-scale morphologies. Slice-wise alignment by SIFT does not ensure maintenance of morphology (indicated by crooked orange line at the platinum coating), whereas alignment by TM does (straight blue line). Orange arrow heads in close-ups indicate deformed slices which could not be properly aligned using translations. This effect showed both in the upper and lower part of the dataset for SIFT and with increasing severity towards the lower part for TM.

In recent years, the development of new electron microscopy technologies allowed the automated serial imaging of entire biological specimens, from cells to model organisms. Amongst these techniques, Focused Ion Beam Scanning Electron Microscopy (FIB-SEM) has emerged as a preferred technology for gathering serial images at isotropic resolution. After volumetric acquisition, an important step for proper visualization and accurate morphometric analysis is the alignment of the image stack along the **z**-axis. However, due to the size and complexity of the data, alignments using simple translations are most commonly used^1,2^ instead of adapting the transformations to the specific type of data. Consequently, the most common algorithms used to find correlation between adjacent slices are alignment by SIFT^3^ and alignment using a template structure matched by cross correlation, also known as template matching (TM). In the specific case of data acquired using the Atlas 5 software^4^, TM is efficiently performed on markings created at the surface of the sample (Fig. 1a). These markings are at a constant position with respect to the flat sample surface. In case of SIFT when only global translations are applied, each slice is aligned to the previous, preserving local morphological properties (a few slices) along the **z**-axis, while disturbing the global shape of objects across long distances. As a consequence, straight objects, such as the sample surface plane, can become crooked in a non-predictable manner (Fig. 2a).

**Figure 3:**
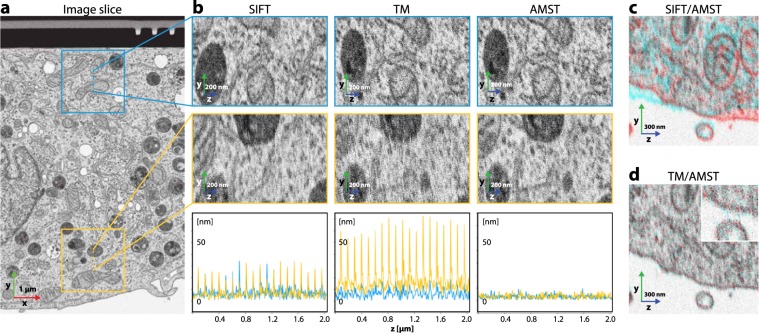
Comparison of alignments by SIFT, TM, and AMST using the proposed evaluation scheme, namely comparison to local SIFT alignments on the HeLa dataset (EMPIAR-10311). (**a**) Image slice which is located in the center of the resliced crops of (**b**). (**b**) Evaluations of the different alignment methods. The microscopy images are resliced crops (**zy**) along the respective dashed line indicated in (**a**). The plots show the displacements an alignment by SIFT performs when run on the areas as indicated by the boxes in (**a**). The distinct regions and their respective displacements are color coded: blue denotes the top part of the dataset, yellow the bottom. The peaks in the alignment error plots for SIFT and TM originate from auto-focus and -stigmation operations during image acquisition. (**c**) Comparison of the AMST and SIFT alignments to show the bias of SIFT along the **z**-axis on the morphology of the sample. (**d**) Comparison of the AMST and TM alignments showing the preservation of the overall morphological structures and the efficacy in correcting for the local image deformations. The inset highlights the difference of the alignments with respect to the maintenance of smooth membranes.

Additionally, for FIB-SEM data, slices can project distorted images when different parts of an imaged cross-section are exposed to different rates of radiation. This effect typically occurs during auto-focus and auto-stigmation operations, which are triggered periodically during a run (Figs. 2 and 3 from b to d). These operations consist of the acquisition of several low resolution images at a specified location within the field of view, which creates non-homogeneous irradiation of the imaging surface. Upon subsequent acquisition of an image slice, differently irradiated regions introduce relative displacements during image formation and hence a distortion that shows an uneven distribution of the pixel size^5,6^. After alignment of the dataset by SIFT, these deformations are especially prominent in the upper and lower parts of the stack, while with TM, the deformations result in a poor alignment quality further away from the template structure, hence at the bottom of the image (Fig. 2).

Several automatic approaches to align volume data have been developed previously. The most recent one applied to serial section transmission electron microscopy (ssTEM), uses elastic deformations in an optimized manner along the **z**-stack^7^. One drawback of this method is that local deformations can become very severe which does not ensure maintenance of morphological properties throughout the image data (Supplementary Fig. S1, Video S1). Moreover, machine learning based segmentation methods, such as membrane prediction by convolutional neural networks (CNNs)^8,9^, can be sensitive to alignment artifacts. For this reason, much finer alignments are necessary for downstream processing.

To overcome the alignment induced drift effect as well as misaligned regions not adjacent to the sample surface, we introduce a novel alignment scheme which we call Alignment to Median Smoothed Template (AMST). This method first creates a template dataset by pre-aligning the stack of images with either TM or SIFT and by smoothing it along the **z**-direction using a median filter. The raw data is then aligned to the template using affine transformations, i.e. linear transformations which include translation, rotation, shearing, and scaling (Fig. 1). Despite the introduction of affine transformations and due to the constraint to the template, our approach does not alter morphological properties of the image data. To evaluate alignments and compare AMST to SIFT and TM, local re-alignments on cropped regions were used, showing a substantial improvement of alignment quality. We demonstrate that better alignment enables a high segmentation quality, which we illustrate by means of an organelle boundary prediction using a CNN.

## Results

Using AMST, we present substantially improved alignments of two FIB-SEM datasets. Both datasets differ in acquisition parameters and sample preparation, highlighting the robustness of the method for different data conditions. Additionally, we investigated the effects of AMST alignment in the downstream analysis for 3D segmentation. We opted for a 3D CNN^10^ and observed that the volume reconstruction of organelle boundaries was substantially improved.

### Limitations of commonly used alignment methods

We set out to measure the limitations of two state-of-the-art alignment methods commonly utilized to align FIB-SEM datasets. First, we tested SIFT (Fiji plugin “Linear Stack Alignment with SIFT”) which is based on the identification of common features on two adjacent images. We also evaluated TM, a common alignment strategy for FIB-SEM, which relies on marks introduced into the platinum layer deposited at the surface of the sample to protect it upon acquisition (Fig. 1a). Such marks serve as reference for the alignment, as they benefit from a high signal to noise ratio, and can be automatically detected by cross-correlation.

On long ranges in the **z**-direction, the results from SIFT and TM alignments differed substantially. Using SIFT, alignments were not maintained over long **z**-distances, as only adjacent image slices are aligned one to the other. As a result, straight structures such as the platinum deposition at the surface of the sample appeared sheared (Fig. 2a). As expected however, when TM was utilized to align the dataset, the flat surface of the sample appeared as a straight horizontal line in a **zy**-projection (Fig. 2b). In this case, the top surface of the sample can be considered perfectly flat, as it corresponds to the surface onto which the cells were growing (here a sapphire cover-slip used as a support for cell culture and compatible with high pressure freezing). Hence, the alignment markings within the coated top of the sample were considered to be straight lines orthogonal to the imaging plane. Using them as a reference for TM leads to a better preservation of the morphological properties of the sample.

In both SIFT and TM, the alignment strategy was restricted to translational movements. However, due to changes in imaging conditions during a standard run with the FIB-SEM, pixel sizes are not constant from one image slice to the next and alignment using translations cannot yield good results. Misalignments were clearly visible at both the top and bottom of the SIFT aligned dataset (Fig. 2a, see orange arrowheads in crops). Although the top of the TM aligned dataset, close to the template, seemed to have negligible alignment errors (Fig. 2b, upper crop), the bottom displayed a clear distortion (Fig. 2b, see orange arrowheads in lower crop). In summary, with SIFT, local misalignments increased with distance from the center of each slice while with TM, local misalignments increased with distance from the surface.

Fixing local misalignments using rotation, scaling, and shearing at the level of the full image or elastic registration in local regions, poses the danger of altering the raw data in undesired ways – commonly resulting in a cascade of transformations that can alter the original shape of the imaged sample (also see Supplementary Fig. S1 and Video S1). Another solution leans on the combination of morphology-preserving TM as pre-alignment in conjunction with the linear stack alignment workflow implemented in IMOD^11^. This method can partially compensate for local misalignments while maintaining the morphology of the sample. However, we found that outlier slices affected the transformation of surrounding slices and introduced rippled membrane surfaces (Supplementary Fig. S2). We thus concluded that none of the current solutions are satisfying for a fine alignment of a FIB-SEM volume, as they fail to fully compensate for local misalignments, or are prone to introducing long range morphological distortions.

### AMST outperforms other alignment procedures

AMST aligns FIB-SEM data and corrects acquisition-induced distortions using a three-step workflow. First, the dataset is pre-aligned using either TM or SIFT by translations (Fig. 1a), depending on the application. Second, the pre-aligned stack is smoothed in the **z**-direction using a median filter (Fig. 1b). This dataset is referred to as the template dataset (Fig. 1c). Third, the template dataset is used as a reference to which the original, non-aligned data is registered slice-wise using affine transformations, (Fig. 1d) thus generating the final result (Fig. 1e).

Affine transformations are linear mappings based solely on translation, rotation, shearing and uniform scaling of images. This implies that parallel lines in the original space will remain parallel in the transformed space. The affine transformations for each data slice were performed using the Elastix toolkit^12,13^ and are formulated as a coordinate mapping from the fixed to the moving image domain (${\bf{T}}:{\Omega }_{F}\subset {{\mathbb{R}}}^{d}\to {\Omega }_{M}\subset {{\mathbb{R}}}^{d}$). In this workflow, the fixed images are the slices of the template dataset and the moving images are those from the raw data. The final registration result ${I}_{M}({{\bf{T}}}_{\mu }({\bf{x}}))$ consists of affine transformations of the raw data slices to the respective template dataset slices which are, for each slice, defined as

1 $${{\bf{T}}}_{\mu }({\bf{x}})=A({\bf{x}}-c)+t+c$$

where *c* is the center of the image, *t* denotes translations, *A* is a non-restricted 2 × 2 transformation matrix, and $\mu ={({a}_{11},{a}_{12},{a}_{21},{a}_{22},{t}_{x},{t}_{y})}^{T}$ is the parameter vector. The parameter vector contains the matrix elements *a*_*ij*_ and the translations in **x** and **y**, *t*_*x*_ and *t*_*y*_, respectively. The affine transformations were solved by iterative optimization (by gradient descent) of the parameter vector *μ* by means of mutual information^14^ defined by Unser *et al*.^15^.

To evaluate the performance of AMST in comparison to SIFT and TM, we cropped two regions of 512 × 512 pixels in the **xy**-plane – one at the top of the dataset and one at the bottom (Fig. 3a, blue and yellow boxes, respectively). For each crop, we ran a local alignment and the displacements were stored. The magnitude of the displacements was used as an indication of the alignment error for each slice (Fig. 3b, bottom row).

In our evaluation, SIFT showed alignment errors for single slices of around 20 to 30 nm at intervals of approximately 0.1 µm in **z**-direction. These misaligned slices appeared as a streaky texture in **zy**-re-slices Figs. 2a and 3b, SIFT, top and middle) and were equally severe in the top and bottom crops. With TM, the alignment at the top part of the slice showed errors of about 10 nm, whereas errors in the bottom part reached up to 60 nm, also yielding a streaky pattern clearly visible in the **zy**-re-slice (Figs. 2b and 3b, TM).

With AMST the error decreased below 10 nm both at the top and bottom of the image with an average displacement error of 3.2 nm which is well below the size of one pixel (5 nm) (Fig. 3b, AMST, bottom). Consequently, the texture of **zy**-re-slices appeared of similar quality compared to an image slice in **xy** (Fig. 3b, AMST, top and middle), thus maintaining the coherence along each axis of the imaged volume.

The main benefits of AMST with TM-pre-alignment are the maintenance of sample morphology and the solving of local misalignments. For direct comparison we overlaid **yz**-re-slices of two different alignment types of the same dataset (cyan and red channel), to reveal their relative **y**-offset in dependence of the **z**-position (Fig. 3c,d). The central slice was set to offset = 0. The overlay of SIFT and AMST showed a strong bias of SIFT in the **z**-direction (Fig. 3c). The same overlay between TM and AMST illustrated that AMST maintained the morphology of the sample coherently along the slices of the stack (Fig. 3d). Here, the slices which contained altered pixel sizes after TM became visible (Fig. 3d, inset).

**Figure 4:**
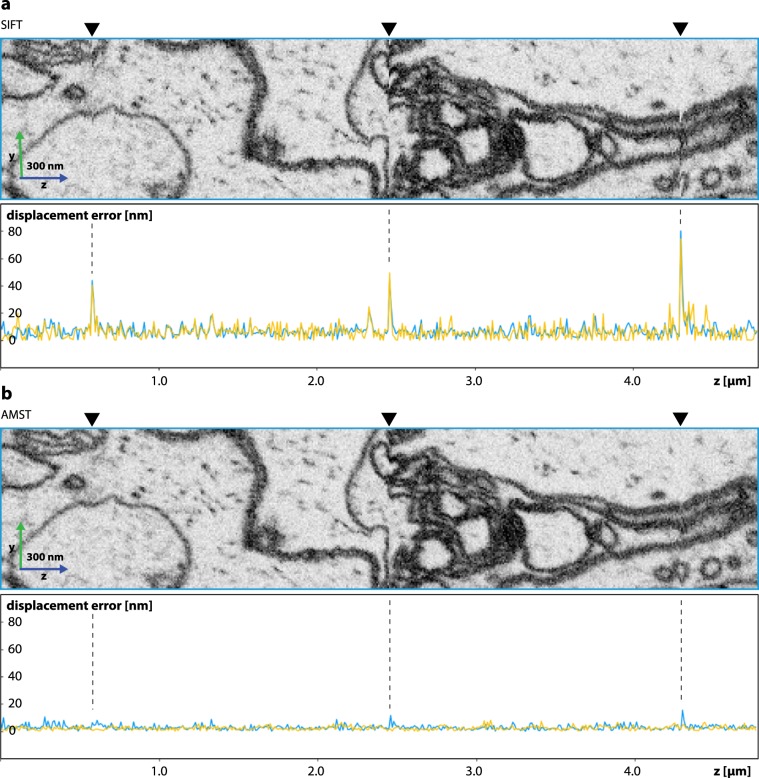
Comparison of alignments by SIFT and AMST when no alignment marking were available in the platinum coating due to embedding by minimal resin technique (*P. dumerilii* dataset, EMPIAR-10310). (**a**) Cropped region from the top of the dataset after alignment by SIFT and the plot of the alignment quality along the same region. The blue and yellow plot correspond to displacement errors in the top and bottom part of the dataset, respectively, as described in Fig. 3. Three major outlier slices are visible in approximately 2 µm distance, (arrowheads and dashed lines) which correspond to auto-focus and auto-stigmation operations of the microscope. (**b**) Illustration of the same region as in (**a**) after alignment by AMST with SIFT serving as pre-alignment. The displacement error of major outlier slices was reduced from up to over 80 nm to an error less than 16 nm. Additionally, the alignment quality generally improved from an average in displacement error of around 7 nm to 2.5 nm.

The pre-alignment step described here can be chosen depending on the sample type. When the surface of the specimen is flat, as it is the case for adherent cells seeded on cover-slips or sapphire disks, TM was the best adapted pre-alignment method. TM maintained the flat sample surface in the resulting alignment as described above. However, in some applications like minimal resin embedded samples^16^, the sample surface is not flat. Here, a feature alignment method like SIFT was used for the pre-alignment (Fig. 4). Whilst, in this case, long range deformations caused by the alignment are hard to evaluate or correct for, local jumps due to image deformation during the run were still present with an average of 7 nm and up to 80 nm for major outliers (Fig. 4a). Using AMST with SIFT pre-alignment, we managed to efficiently abate the amplitude of local displacements in outlier slices with a reduction to less than 15 nm and an average error of 2.5 nm (Fig. 4b).

Altogether, these results showed that the AMST alignment method preforms significantly better compared to routinely used alignments (TM or SIFT) for preserving the anatomical integrity of specimens acquired by FIB-SEM.

### Automated 3D membrane reconstruction improves on AMST processed datasets

To illustrate the impact of a high-quality alignment on downstream processing of the data we set up a segmentation pipeline to predict organelle boundaries and plasma membrane using the dataset shown in Figs. 2 and 3. We used a 3D U-Net^10^ with batch normalization and L2 regularization.

**Figure 5:**
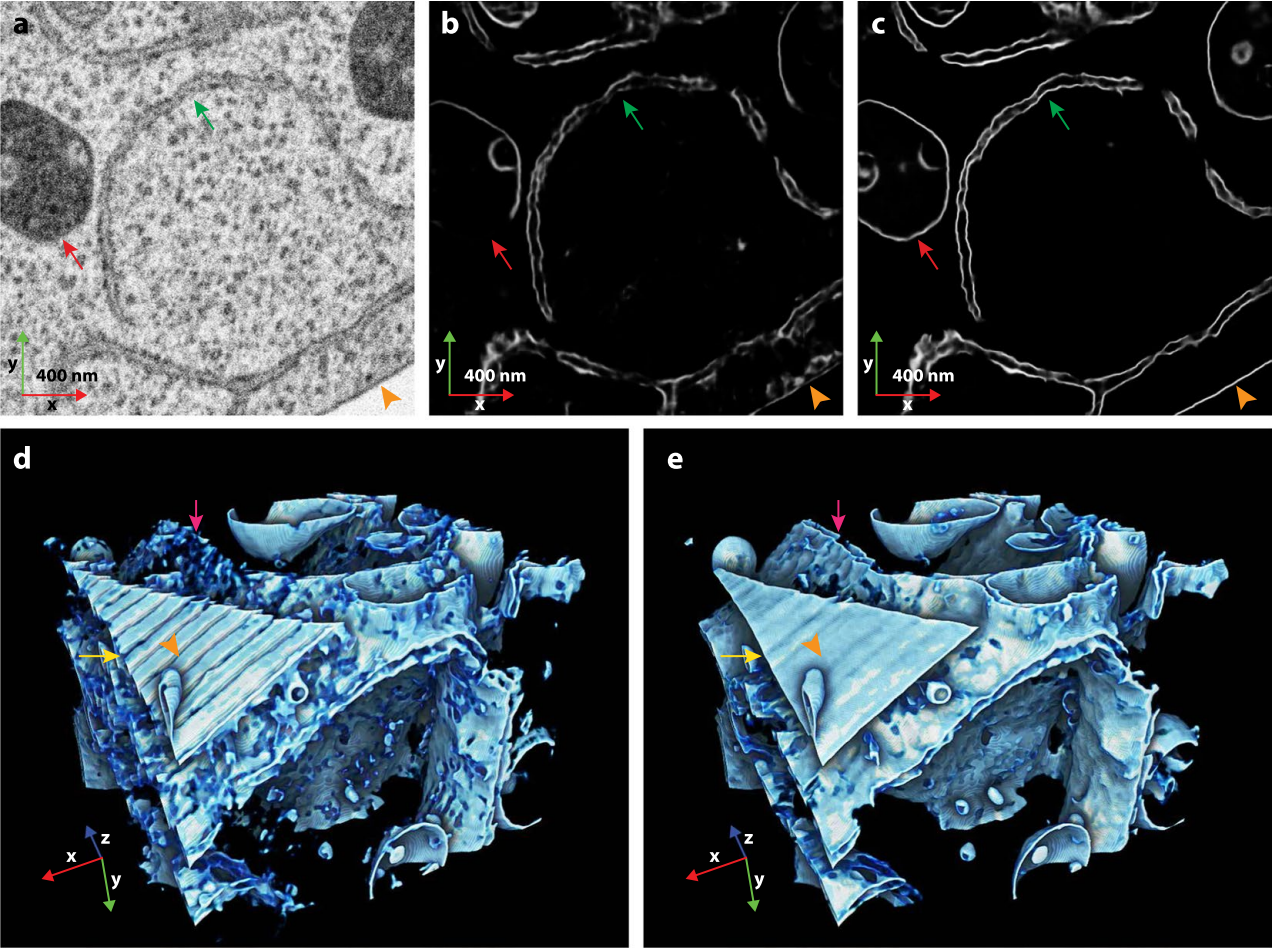
Qualitative comparison of 3D U-Net membrane reconstructions using TM or AMST aligned datasets (subsets from aligned HeLa dataset, EMPIAR-10311). (**a**) Raw data slice of the test set. (**b,c**) Membrane reconstructions of the same slice from TM and AMST aligned datasets, respectively. Red and green arrows indicate organelle boundaries that were substantially improved upon alignment with AMST. (**d,e**) 3D renderings of a sub-volume containing the predictions shown in (**b,c**), respectively. Membrane reconstruction quality was substantially improved when using AMST aligned data. Jumps in the data alignment as found in the TM dataset, frequently disrupted the membrane prediction and thus membrane connectivities. The 3D renderings were oriented such that a stretch of the plasma membrane is visible in the front. The orange arrow heads (**b–e**) point to the same location in identical direction parallel to the image slices. The yellow arrows highlight the plasma membrane, magenta arrows an ER sheet.

Our segmentation results show a striking difference of the membrane prediction quality between TM and AMST. On the validation set, for the membrane class in TM and AMST aligned datasets, the dice coefficients were 0.25 and 0.34, respectively, showing a substantial improvement of the segmentation on the validation set. However, the validation set was directly adjacent to the training set, so we additionally selected a distant region at the plasma membrane of the cell as a test set. Here, qualitative analysis showed that membranes of organelles (endoplasmic reticulum and endosome) were frequently lost for TM, while successfully reconstructed on the AMST dataset (Fig. 5a–c, red arrows and Supplementary Video S2). This was further highlighted in the membranes of the ER which appeared fuzzy in the reconstruction from the TM dataset, while being correctly resolved from the AMST dataset (Fig. 5a–c, green arrows and Supplementary Video S2). A quantitative assessment of the segmentation was measured by comparing the automated segmentation to a manually curated ground truth using intersection over union. The AMST alignment indeed led to an improved segmentation efficiency (IoU_TM_ = 0.37 and IoU_AMST_ = 0.47, Supplementary Fig. S3).

3D rendering also showed the quality difference between membrane reconstructions originating either from TM or AMST datasets (Fig. 5d,e). For illustration purposes, the 3D rendering was oriented such that a section of the plasma membrane is visible in the foreground. The orange arrow head points to the same location and in the same direction parallel to the image slices for all subfigures (Fig. 5b–e). On the reconstruction from TM aligned dataset, the pattern created by the major outlier slices (Figs. 2 and 3) broke the connectivity of the plasma membrane (Fig. 5d, yellow arrow). Also, some areas of ER membrane were completely lost (Fig. 5d, magenta arrow). The reconstruction performed on the AMST aligned dataset fixed these issues, the plasma membrane was nicely reconstructed as a smoother and continuous sheet (Fig. 5e, yellow arrow) and the formerly missing ER stretches were successfully recovered (Fig. 5e, magenta arrow).

## Discussion

The interest for ultrastructural volume imaging is growing in Biology. When using scanning electron microscopes, all methods are based on imaging the specimen one slice after another. Specialized methods are thus crucial to preserve or restore the integrity of the volume by correcting local distortions and keeping coherence along the **z**-axis. Moreover, well aligned image stacks significantly improve the fidelity of the visualization as well as the segmentation accuracy and quantification. For the correction of local distortions and the positive effect on downstream analysis AMST presents a novel alignment strategy developed for FIB-SEM data volumes.

On samples with a flat surface AMST benefits from a pre-alignment by TM, which best maintains morphological properties of the sample. It then solves local misalignments, caused by distortions occurring during image acquisition, by re-alignment of images based on a morphology-preserving median smoothed template using affine transformations. Affine transformations include rotation, shearing and scaling, usually resolved using interpolation, which, in extreme cases, can cause blurring in transformed images. However, the actual amount of blurring introduced by these transformations in AMST is minimal as the median smoothed template is computed over isotropic or nearly isotropic datasets and the transformations are restricted to a level of a few pixels.

Additionally, we show the impact an improved alignment has on automatic segmentation of organelles and plasma membranes. Although it is well known that deep learning networks are resilient to errors in alignment, the ability of the CNN to predict improved substantially upon re-alignment. This enabled a more faithful recovery of structures such as ER membrane sheets. Also, smooth membranes, such as the plasma membrane, showed substantially less artifacts and could, on the re-aligned dataset, be reconstructed as one continuous plane.

AMST is a tool developed for fine tuning an aligned dataset to correct for alignment errors originating from a random alteration in pixel sizes, where the deformations of adjacent slices are non-correlated. Consequently, the AMST re-alignment step cannot curate alignment errors on larger scales such as a drift effect as described for SIFT aligned datasets. This kind of alignment error should be solved at the level of the pre-alignment. Defective or missing slices are currently not specifically handled, as, due to the median filtering, single aberrant slices do not have a negative effect on the final result. A further improvement of the workflow could include a more specifically adapted pre-alignment which detects and curates missing or damaged slices.

Avoiding larger scale alignment errors is especially difficult due to the lack of **z**-references. In FIB-SEM datasets originating from cells growing on a flat cover-slip, we propose that the most sensible approach to align along **z** is to consider the top of the sample with the platinum coat as a flat surface without any type of slope or variation. An uneven sample surface, for example due to dust particles, can introduce a morphological error in affected slices. Since this usually affects multiple consecutive slices, AMST will not correct for this. In these particular cases, a manual correction is needed for the affected slices as part of the pre-alignment step and before running AMST. If a general slope in the surface of the sample is known, a possible improvement could be to introduce the slope artificially within an extra step using an affine transform after applying AMST.

In addition, the introduction of side fiducial marks can be used to measure thickness variations^17^. FIB-SEM datasets show a certain degree of inaccuracy between the thickness of each section at a nanometer scale, which can vary up to 50% of the expected **z**-thickness, altering the final voxel size^18^. The reasons attributed to these fluctuations are multiple: the nature of sample materials which can change during the FIB slicing, thermal fluctuations or charge balance stabilization between the ion and the SEM beams. AMST is focused on correcting SEM distortions in **x**- and **y**-direction while maintaining morphology along the **z**-axis (based on a pre-alignment). As it is not designed to compensate for uneven voxel sizes, future implementations could extend the present alignment functionality to 3D. In conclusion, we believe AMST is a straight forward solution that could be generalized to aligning any datasets produced by FIB-SEMs, paving the way for more accurate volume segmentation and analysis.

## Methods

### Sample preparation and imaging

The data in Figs. 1–3 and 5 show one cell from a cell culture of GalNAc-T2–GFP HeLa cells^19^ that were grown on a sapphire disk (Wohlwend) and high-pressure frozen (HPM 010, ABRA Fluid AG). The method for freeze substitution (EM AFS, Leica) and Epon embedding was adapted from Walther *et al*. and Villinger *et al*.^20,21^. The freeze substitution medium contained 1% osmium tetroxide, 0.5% uranyl acetate and 5% water diluted in acetone. The temperature was raised from −90 °C to 20 °C within 22 hours (5 °C per hour). The samples were then kept at 20 °C for an additional hour and then rinsed several times with pure acetone. Epon infiltration was performed in the microwave (Pelco BioWave, Ted Pella) in multiple steps (all were 3 minutes at 250 W with vacuum on) while gradually increasing the resin concentration (1 × 50% resin/acetone, 3 × 100% resin). The resin was polymerized at 60 °C for 48 hours. Samples were mounted onto SEM stubs with a carbon sticker, surrounded with silver paint and sputter coated with gold (Quorum Q150R S).

*Platynereis dumerilii* (Fig. 4) were raised in natural sea water under a 16 hour light and 8 hour dark cycle at 18 °C in an established culture at the European Molecular Biology Laboratory in Heidelberg. At six days post fertilization, *P. dumerilii* were anaesthetized (in a 1:1 solution of 7.5% MgCl_2_ and sea water for 10 minutes) and fixed in 2% formaldehyde in 0.1 M cacodylate buffer. Immediately followed by a second step of fixation in 2.5% glutaraldehyde in 0.1 M cacodylate buffer. Both fixation steps were completed within a microwave (Pelco Biowave, Ted Pella) at a regime of two 14 minute cycles of 2 minutes on/2 minutes off, at 150 W under vacuum. The method for *en bloc* staining for *P. dumerilii* was adapted from Hua *et al*.^22^. After fixation samples were rinsed with 0.1 M cacodylate buffer and post fixed with 2% osmium tetroxide in 0.1 M cacodylate buffer. Following this, samples were immersed in 2.5% potassium ferrocyanide also buffered with 0.1 M cacodylate, then 1% thiocarbohydrazide (aqueous) and lastly 2% osmium tetroxide (aqueous). Each step was performed in the microwave and all steps excluding the first osmium tetroxide were followed by thorough rinsing with distilled water. Samples were dehydrated with increasing concentrations of ethanol (20, 50, 70, 90, 3 × 100% in the microwave each for 40 s at 250 W, no vacuum) and infiltrated with durcupan resin (25, 50, 75, 2 × 100% in microwave each for 3 minutes at 250 W with vacuum on plus one step of 100% overnight on the bench). Samples were embedded using the minimal resin technique^16^. After polymerization at 60 °C, they were mounted onto SEM stubs with silver epoxy and sputter coated with gold (Quorum Q150R S).

FIB-SEM image acquisition was performed with the Zeiss Auriga 60 or a Zeiss Crossbeam 540 using the Atlas 5 software (FIBICs, Carl Zeiss Microscopy). We used the sample preparation workflow of Atlas 3D in the software package containing the following steps: (i) Deposition of a 1 µm of platinum coating onto the sample surface at the region of interest using the gas injection system (GIS), (ii) FIB etching of autotune marks into the platinum followed by an additional insulator coating to fill the marks, and (iii) exposition of the sample imaging surface by FIB-milling of an initial trench. For both datasets the images were acquired at 1.5 kV with the energy-selective back-scattered electron (EsB) detector with a grid voltage of 1100 V, analytical mode at a 700 pA current. The dwell time was 4 µs/10 µs, with line average of 1. For the HeLa dataset images had a 5 nm **xy** pixel size and 8 nm slicing thickness in the **z**-direction and for the *P. dumerilii* dataset, images had a 10 nm **xy** pixel size and 10 nm slicing thickness. During the 3D data acquisition the FIB was operated at 1 nA/1.5 nA with the SEM and the FIB operating simultaneously^23^. In the HeLa cell dataset the tracking was active for the first half, but then failed. For the *P. dumerilii* there was no form of **z**-slice thickness tracking performed.

### Alignment to median smoothed template (AMST)

The AMST workflow is composed of three major steps, namely (i) generation of a pre-alignment, (ii) median-smoothing of the pre-alignment yielding a template dataset, and (iii) alignment of the raw data to the template dataset, described in the following in detail:

(i) When available, alignment markings in the platinum coating were used. These were assumed to be located at the same position within each slice of the image data and, consequently, served as a strong reference to guide the TM to generate a pre-alignment. We use the Fiji plugin developed by Tseng (https://sites.google.com/site/qingzongtseng/template-matching-ij-plugin) where the template is matched with normalized correlation coefficient. To obtain best possible results, subpixel displacements with bilinear interpolation to place slices according to the template position were used.
  If no alignment markings were available, alignment by SIFT (“Linear stack alignment with SIFT” in Fiji^3^) was used while only allowing translational movement alongside the default parameter set including an initial Gaussian blur of $\sigma =1.6\,{\rm{px}}$.
(ii) The resulting aligned data was then smoothed with a median filter (filter size of 15 pixels along the **z**-axis, no filtering along the **x**- and **y**-axes) to generate the template dataset. By this means, information from slices that were altered in pixel size with respect to their neighbors (due to aberrations or charging in the image acquisition process) was removed.
(iii) For the final step, the Elastix toolkit^12,13^ was used to align the raw data to the template dataset. The raw data was mapped to the template using affine transformations, meaning that the image could be translated, rotated, scaled, and sheared. The registration was solved in 200 iterations using gradient descent as introduced by Klein *et al*.^24^ and by Mutual Information^14^ serving as similarity measure. As, in practice, Elastix requires moving and fixed images to already be in close proximity, we added an optional translation step (SIFT implementation from the Silx python package^3^, after an initial Gaussian blurring with $\sigma =1.6\,{\rm{px}}$), hereby bringing the raw data close to the template before applying the affine transformation with Elastix.

The current implementation in python can be found on GitHub (https://github.com/jhennies/amst). The runtime of the workflow depends linearly on the number of slices. The full workflow runs on a desktop computer on CPU (median smoothing and affine registration) and GPU (the optional SIFT step) within several hours for an average-sized FIB-SEM dataset (approximately 1 hour for a dataset with approximately $6000\times 3000$ pixel section size and 512 slices using a maximum 12 CPU cores; also see Supplementary Table S1). The components (median smoothing and re-alignment) are individually parallelized over the image slices, resulting in increased performance with additional CPU cores available. Memory consumption is not limiting, as only the currently processed image slices are loaded from disk.

### Evaluation of alignments

FIB-SEM data, although showing aberrations with respect to pixel sizes on a larger scale, was assumed to have homogeneous pixel sizes on a local scale (e.g., $512\times 512$ pixels). To evaluate the quality of an alignment of a full dataset, first regions of 512 × 512 pixels were cropped along a sensible **z**-range using the aligned data. The two regions were chosen according to the following criteria: (i) A region contains features that can be used to align over the full window and in all its **z**-slices; (ii) the regions are as distant as possible to each other such that they represent different parts of the full dataset (e.g., one region near the top and one near to bottom of the dataset, Fig. 2a,b). To fulfil both (i) and (ii) most effectively, the data usually needed to be split into subsets along the **z**-stack. Subsequently, we used SIFT to compute displacements that would locally optimize the alignment, comparing one slice to the previous. The absolute value of this displacement was used as quality measurement, as it represents the local error of the overall alignment. We plotted the error, expressed in nanometers, over the image slices (**z**-axis). Additionally, for qualitative alignment, we overlaid **z**-re-slices of SIFT and AMST as well as TM and AMST results, respectively. Here we put TM/SIFT in the red channel and AMST in the cyan channel such that differences in the respective alignments become visible.

### Automated 3D membrane reconstruction

Automated 3D membrane reconstruction was performed using a CNN implemented in Keras^25^ with Tensor Flow backend^26^ with a 3D U-Net architecture as described in Çiçek *et al*.^10^ and an input volume size of 64 × 64 × 64 voxels. The network was trained in batches of one volume and by using batch normalization. Regularization was performed using L2 regularization with the regularization parameter $\lambda ={10}^{-5}$. Our annotated data consisted of a 512 × 512 × 64 voxel densely annotated subset of the HeLa dataset which we split into training data (3/4) and validation data (1/4), individually for the TM and AMST aligned datasets. The model was trained for 100 epochs with 100 steps per epoch until convergence and the epoch after which the model performed best on the validation data was selected. The results shown were computed on two 512 × 512 × 512 voxels test sets, of the same region for TM and AMST respectively, by splitting the test sets into 64 × 64 × 64 cubes with an overlap of 32 voxels in each dimension. The area for the test sets was chosen such that it was distant to the training and validation data as well as distant to the markings in the insulator coating used for alignment. 3D rendering of the stitched probability map of the test sets was performed using Drishti^27^.

## Supplementary information

Supplementary video S1: FIB-SEM dataset alignment with elastic deformations.

Supplementary video S2: Impact of AMST on the quality of automated membrane predictions.

Supplementary information .

## Data Availability

The datasets used for method development and shown in this manuscript are available in the EMPIAR repository: **HeLa**: https://www.ebi.ac.uk/pdbe/emdb/empiar/entry/10311/**, P. dumerilii**: https://www.ebi.ac.uk/pdbe/emdb/empiar/entry/10310/
