# Supplementary material for: AMST: Alignment to Median Smoothed Template for Focused Ion Beam Scanning Electron Microscopy Image Stacks
Source: Sci Rep. 2020 Feb 6;10:2004. doi: 10.1038/s41598-020-58736-7 (PMC7004979; doi:10.1038/s41598-020-58736-7)
Supplement: Supplementary file 3 — Supplementary information . [file 41598_2020_58736_MOESM3_ESM.pdf]

Supplementary to AMST: Alignment to Median Smoothed Template for Focused Ion Beam Scanning Electron Microscopy Image Stacks

Julian Hennies[1,2,*], José Miguel Serra Lleti[1], Nicole L. Schieber[1], Rachel M. Templin[1], Anna M. Steyer[3], and Yannick Schwab[1,*]

[1]European Molecular Biology Laboratory (EMBL), Cell Biology and Biophysics Unit, Heidelberg, 69117, Germany
[2]Collaboration for joint PhD degree between EMBL and Heidelberg University, Faculty of Biosciences
[3]Max Planck Institute for Experimental Medicine, Electron Microscopy Core Unit, Department of Neurogenetics, 37075 Göttingen, Germany
[*]hennies@embl.de, schwab@embl.de

Elastix parameter optimization

We optimized Elastix parameters specifically for the use of AMST on the two datasets (HeLa and *P. dumerilii*) to obtain both a good computation performance as well as high quality of the alignment. We started off from the default parameter set for affine transformations (`http://elastix.bigr.nl/wiki/images/c/c5/Parameters_Affine.txt`), then different parameters were progressively tested by trial and error. A decrease in computational time was achieved by decreasing the number of resolutions which is used for finding the right transformation from 4 to 3 (NumberOfResolutions = 3) with downsampling of 8, 3 and 1, respectively (ImagePyramidSchedule = $(8,8,3,3,1,1)$). Additionally, the number of iterations per resolution level was set to NumberOfIterations = 200. To enable fast convergence, we increased the maximum step length of the gradient descent to MaximumStepSize = $(4,2,1)$, i.e. a maximum of four pixels in the first, of two pixels in the second and one pixel in the last resolution level (the default is one pixel for any resolution). A general improvement of alignment quality was achieved by setting AutomaticScalesEstimation = **False** and ImageSampler = ”RandomCoordinate”.

Alignment with elastic deformations

In this work we opted for the use of affine transformations over elastic deformations. Elastic deformations can, in principle, model any deformation that could occur in image data. However, the danger of unrealistic local and large-scale deformations is high. We ran an alignment with elastic deformations on the HeLa dataset using an implementation described in Saalfeld et al.[1] which is implemented in the Fiji plugin TrakEM2[2]. We used the method to optimize an alignment obtained by template matching (TM) to maintain sample morphology as good as possible and for better comparison to the results from AMST. For alignment in TrakEM2, we used the default parameter set and optimized the maximum_stretch parameter (default maximum_stretch = 2000). A reduction to maximum_stretch = 8 yielded improved local alignment quality as well as a reduction in deformation. Increasing the number of neighborhood slices to which each slice is compared did not yield a better alignment quality in terms of local alignment error. We selected the best performing set for qualitative and quantitative analysis (supplementary Fig. S1 and Video S1) for comparison with AMST.

Serial Stack Alignment with IMOD

We compared the performance of AMST against IMOD's[3] Serial Section Alignment (SSA) implemented within the Etomo user interface (the documentation of the alignment workflow can be found here: `https://bio3d.colorado.edu/imod/doc/serialalign.html`). SSA implements the Xfalign function for automatic stack alignment. Optionally the tiltXcorr function can be used to obtain an initial alignment in case slices are not pre aligned. However, we used SSA to correct for local misalignments of a previously TM aligned dataset which leads to best possible preservation of sample morphology. Results from both IMOD and AMST show identical larger scale morphology (compare supplementary Fig. S2 a to b and c to d, respectively). In practice, we loaded the dataset (previously converted to the mrc format using IMOD's tif2mrc function), activated “find initial shifts with cross-correlation” (runs tiltxcorr) and “search for full linear translations” (enables affine transformations), and ran both the “Initial auto alignment” as well as the “Refine with auto alignment”. After processing, we generated the aligned stack in the “Local fitting (retain trends)” mode, which maintained the original morphology of the data.

Computational times

Computational times were measured on the HeLa dataset using a laptop with Intel® core™ i7-9750H CPU (12 threads at 2.60 GHz), NVidia GeForce RTX 2070 and 64 GB RAM running Ubuntu 18.04.3 LTS. For each method we performed three measurements (N = 3) using a different 50 slice subset of the data each time (first: slices 0 to 49; second: slices 50 to 99; third: slices 100 to 149).

For 50 slices	AMST	SSA	TrakEM2
Mean	317 ± 4.2	249 ± 88	109 ± 2.1

Table S1. Computational times for AMST with improved Elastix parameters, Serial Section Alignment from IMOD, and alignment with elastic deformations from TrakEM2.

Organelle boundary predictions

Boundary predictions were computed using a 3D U-Net (Fig. 5, Video S2). We quantified the boundary prediction by means of Intersection over Union (Fig. S3a). Here, we compared to a 256 voxel cubes subset of the results to a manually curated ground truth (supplementary Fig. S3b). We binarized the respective results for membrane predictions on AMST and TM aligned datasets using multiple thresholds. Sample slices for the respective best thresholds are shown in supplementary Fig S3c and d, for AMST and TM, respectively. The best results for intersection over union were found for TM with threshold = 0.3 and $IoU_{TM} = 0.37$ and for AMST with threshold = 0.4 with $IoU_{AMST} = 0.47$.

References

1. Saalfeld, S., Fetter, R., Cardona, A. & Tomancak, P. Elastic volume reconstruction from series of ultra-thin microscopy sections. *Nat. Methods* **9**, 717–720, DOI: 10.1038/nmeth.2072 (2012).

2. Cardona, A. *et al.* TrakEM2 Software for Neural Circuit Reconstruction. *PLoS ONE* **7**, e38011, DOI: 10.1371/journal.pone.0038011 (2012).

3. Kremer, J. R., Mastronarde, D. N. & McIntosh, J. R. Computer Visualization of Three-Dimensional Image Data Using IMOD. *J. Struct. Biol.* **116**, 71–76, DOI: 10.1006/JSBI.1996.0013 (1996).

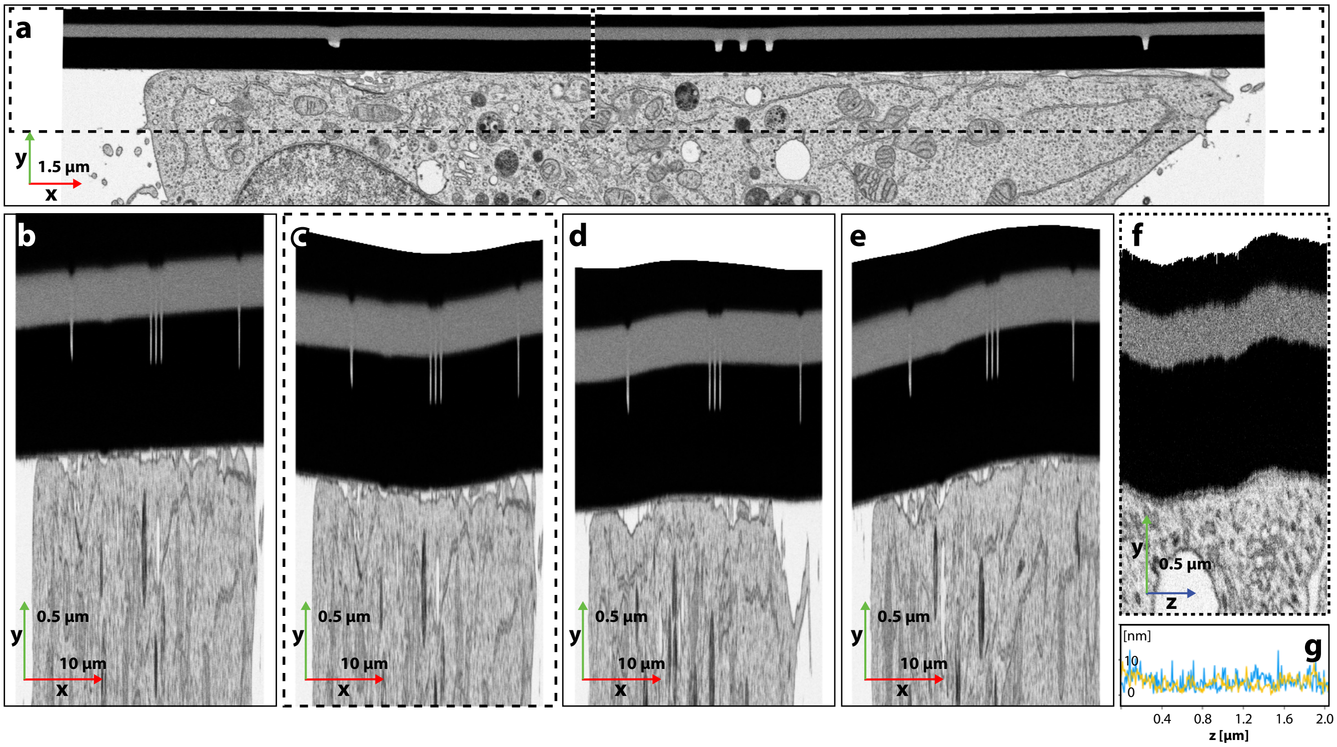

Figure S1. Alignment using elastic deformations. From a) to d) x-y slices from the Hela dataset (EMPIAR-10311), where (a) shows the native 5 nm pixel size. b) to e) depicts the region marked in dashed lines for different slices scaled in the x-direction by 0.05 to better illustrate the deformations of the elastic alignment. b) The original image slice previous to any alignment, c) to e) image slices after elastic alignments. The dashed box in (a) corresponds to the same data shown in (c). f) Re-slice of the data (corresponding to dashed line in (a)) illustrating the deformations in the **z**-direction. f) and g) We measured alignment quality with the same scheme as illustrated in Fig. 3. The local alignment quality was on average well below 10 nm (i.e. two pixels), with some slices not properly corrected (e.g. slices from Fig. 2 and Fig. 3).

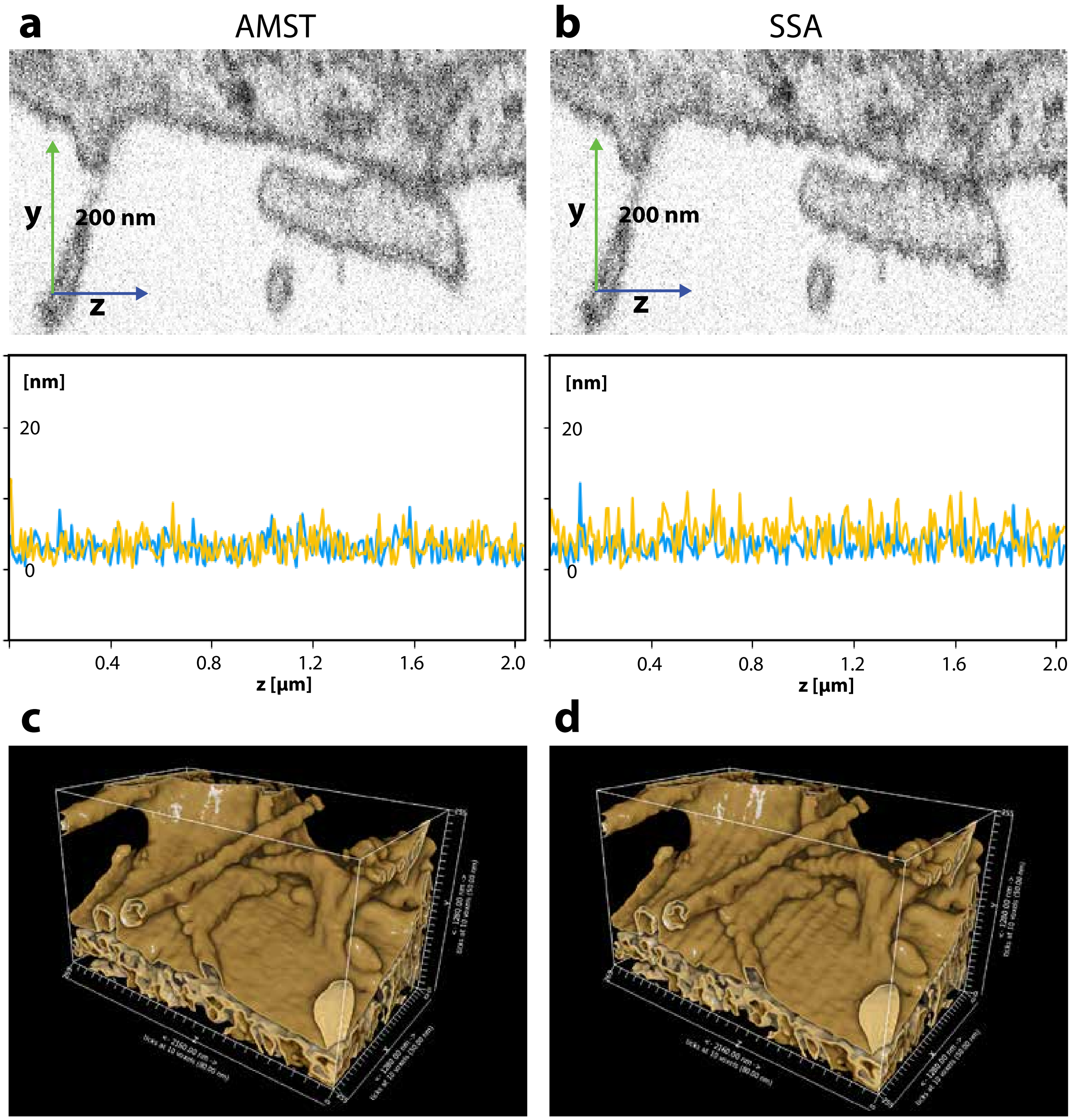

Figure S2. Comparison of a) AMST and b) SSA using the HeLa dataset (EMPIAR-10311). The images in (a) and (b) show an **yz**-reslice in the region of the plasma membrane (top) illustrating a rippled characteristic of the plasma membrane when aligned with SSA. The local alignment error, computed as described for Fig. 3 and supplementary Fig. S1, was similar for both approaches (bottom). 3D rendering of 512 x 512 x 512 voxel subsets of c) AMST and d) SSA results also show the ripples in the plasma membrane introduced by SSA. 3D renderings were performed in Drishti using the respective aligned dataset pre-smoothed by 3D Gaussian smoothing ($\sigma = 2$).

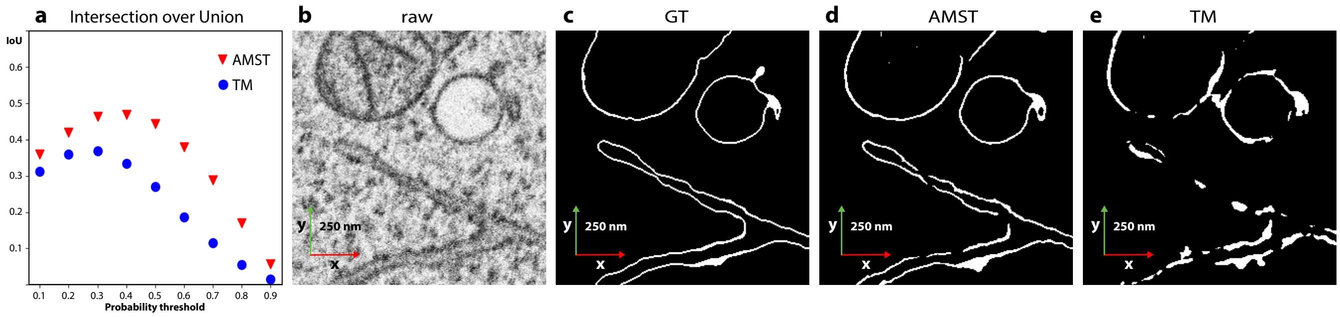

Figure S3. Quantification of membrane prediction results using Intersection over Union (IoU) (HeLa dataset, EMPIAR-10311). a) IoU of AMST and TM boundary segmentation result against a manually proof-read ground truth. The prediction probability maps were binarized with thresholds between 0.1 and 0.9 (**x**-axis) and IoU was computed for each. Using AMST, the membrane prediction showed the best performance at threshold = 0.4 with $IoU_{AMST} = 0.47$; with TM, the IoU was best at threshold = 0.3 with $IoU_{TM} = 0.37$. b) Example slice of the raw data. c) Manually proof-read ground truth. d) Thresholded membrane prediction of sample slice for AMST aligned dataset at threshold = 0.4. e) Thresholded membrane prediction for TM at threshold = 0.3. Images in (b), (c), (d) and (e) show the same area.
